# RE-EXAMINING THE ISSUE OF FALSE POSITIVES IN THE ERA OF BIG DATA AND HIGH-FREQUENCY HEALTH MEASUREMENT TECHNOLOGIES

**DOI:** 10.1093/geroni/igae098.1817

**Published:** 2024-12-31

**Authors:** Terry Haines, Richard Beare, Velandai Srikanth

**Affiliations:** Monash University, Melbourne, Victoria, Australia; Monash University, Melbourne, Victoria, Australia; Monash University / Peninsula Health, Frankston, Victoria, Australia

## Abstract

Technologies that monitor patient health parameters create abundance of data, enabling multiple comparisons to be easily undertaken within the same dataset. This increases the risk of spurious significant findings being reported (type 1 error). Previous work has identified this problem for situations where there is i) one explanatory and multiple outcome variables, 2) multiple explanatory and one outcome variable, and 3) multiple explanatory and multiple outcome variables. We introduce discussions of where there is one explanatory and one outcome variable, but multiple measurement points permitting multiple ways to “slice” data for analysis. We conducted 1,000 Monte Carlo simulations of a pre-post intervention study with additional parallel control site, with each phase having 24 data points per site. We analysed the data as runs of a pre-post study (period), a post-only parallel control (site) and as the full design examining a period-by-site interaction effect. We analysed the full 24 data points per period / site, then 20, 16, 12 using data adjacent to the pre-post transition time point within each run. We then repeated this procedure with 1000x these numbers of data points. We calculated the family-wise type 1 error rate for each analysis run. The cumulative type 1 error rate across all runs based on the 24 data point set was 26.8%, while the rate was 21.9% for runs based on the 24,000 data point set. This means an analyst with enough data can spuriously manufacture a significant finding for one in four to five studies.

